# The Sustained Influence of an Error on Future Decision-Making

**DOI:** 10.3389/fpsyg.2017.01077

**Published:** 2017-06-29

**Authors:** Björn C. Schiffler, Sara L. Bengtsson, Daniel Lundqvist

**Affiliations:** ^1^Department of Clinical Neuroscience, Karolinska InstitutetStockholm, Sweden; ^2^NatMEG, Department of Clinical Neuroscience, Karolinska InstitutetStockholm, Sweden

**Keywords:** post-error slowing, drift diffusion model, visual search, cognitive control, error monitoring, facial emotional stimuli

## Abstract

Post-error slowing (PES) is consistently observed in decision-making tasks after negative feedback. Yet, findings are inconclusive as to whether PES supports performance accuracy. We addressed the role of PES by employing drift diffusion modeling which enabled us to investigate latent processes of reaction times and accuracy on a large-scale dataset (>5,800 participants) of a visual search experiment with emotional face stimuli. In our experiment, post-error trials were characterized by both adaptive and non-adaptive decision processes. An adaptive increase in participants’ response threshold was sustained over several trials post-error. Contrarily, an initial decrease in evidence accumulation rate, followed by an increase on the subsequent trials, indicates a momentary distraction of task-relevant attention and resulted in an initial accuracy drop. Higher values of decision threshold and evidence accumulation on the post-error trial were associated with higher accuracy on subsequent trials which further gives credence to these parameters’ role in post-error adaptation. Finally, the evidence accumulation rate post-error decreased when the error trial presented angry faces, a finding suggesting that the post-error decision can be influenced by the error context. In conclusion, we demonstrate that error-related response adaptations are multi-component processes that change dynamically over several trials post-error.

## Introduction

A typical response to errors in decision making tasks is an increase in response time on trials following the error (Rabbitt, 1969; Laming, 1979). This so-called post-error slowing (PES) has traditionally been attributed to indicate a cognitive control process (Botvinick et al., 2001; Ridderinkhof et al., 2004), ensuring more cautious decision making. However, whether PES is indeed a beneficial process linked to a post-error improvement in accuracy in the sense of a speed-accuracy trade-off, as predicted by the cognitive control account (Laming, 1979; Bogacz et al., 2010), a by-product of a re-orienting process initiated by the error (Notebaert et al., 2009; Houtman and Notebaert, 2013), or a detrimental process reflecting capacity limitations in response monitoring (Jentzsch and Dudschig, 2009) is not clear (as discussed in reviews by Danielmeier and Ullsperger, 2011; Ullsperger et al., 2014). For instance, in a visual search task, Steinhauser et al. (2017) found operation specific post-error adjustments in the same source as the faulty cognitive process, which lends support for cognitive (and thus adaptive) control processes playing a role post-error. And in a modified Stroop task, Hajcak et al. (2003) found a relation between he duration of the PES and post-error accuracy, even after inter-trial intervals of up to 5 s. On the other hand, Notebaert et al. (2009) observed that participants were slowing after both errors and corrects if these were infrequent. Notebaert et al. (2009) proposed that it is instead the commonly low frequency of errors which captures attentional resources. Another explanation for a non-functional account of PES was given by Jentzsch and Dudschig (2009) who suggested that the post-error monitoring process takes up limited central resources and therefore inhibits decision processes subsequent to the error.

Beyond the immediate error-correction on the first trial after an error, there are indications that error monitoring processes may improve accuracy on responses that occur later in time (Hester et al., 2007, 2008, 2009; Klein et al., 2007; Schiffler et al., 2016). For instance, Hester and colleagues demonstrated that posterior medial frontal cortex activity both on error (Hester et al., 2009) and post-error trials (Hester et al., 2007) was associated with decision accuracy several trials later. Further, a recent study showed that memory-reliant PES may benefit learning in a reinforcement learning context (Schiffler et al., 2016). Here, the slowing was related to stimulus specific errors that occurred on average 22 s earlier, which demonstrates that post-error response time adaptations can serve a function in learning on a longer timescale.

A recently proposed framework by Ullsperger and Danielmeier (2016) could potentially link the conflicting accounts of PES. This account suggests, based in part on results from a motion discrimination task (Purcell and Kiani, 2016) that an error invokes a two-component adaptation process: The post-error reaction is marked by (a) an increase in the threshold to commit a decision on the one hand and (b) a decreased rate of sensory evidence accumulation on the other hand. In practice, this means that the reaction time (RT) on the first trial after an error is slower, but the time is momentarily used less efficiently with regard to gathering necessary task-specific information. According to this proposal, task selective attention should increase over the trials following the first post-error trial and thereby increase performance accuracy. This stipulation is reinforced by recent results from a dual task paradigm, showing both control and interference components in PES (Steinhauser et al., 2016).

The unifying framework by Ullsperger and Danielmeier (2016) is further supported by results from drift diffusion modeling. Drift diffusion models have become increasingly instrumental in studying the latent decision process underlying RT adaptations (for recent reviews, see Forstmann et al., 2016; Ratcliff et al., 2016). These models rely on the idea that during decision making in two-alternative forced choice tasks, evidence is accumulated in a noisy fashion toward one or the other response alternative presented until a decision boundary is reached (Ratcliff and McKoon, 2008). When studying response speed adaptations on immediate post-error trials, Dutilh et al. (2012b) found that PES reflected an increase in the distance between the two decision boundaries, the so called *decision threshold*, which indicates increased response caution. In addition, Purcell and Kiani (2016) also demonstrated a decrease in *evidence accumulation* related to PES, suggesting an attentional decoupling from the current task on the first post-error trial. Changes in *non-decision time* in diffusion models can be attributed to afferent and efferent processes relative to the decision. Thus, the non-decision time entails both early visual processing, e.g., time until stimulus reaches the retina and feature extraction, and motor execution of the decision (Ratcliff and McKoon, 2008). Previously, reductions in non-decision time have particularly been found when response speed was emphasized (Rinkenauer et al., 2004; Mulder et al., 2010).

A possible reason as to why evidence accumulation rate decreases and response time slows at a time apparently calling for alertness and efficient cognition, may be that error monitoring is influenced by the emotional content on the error trial (Wiswede et al., 2009; Caudek et al., 2015; Maier et al., 2016) and hence involves emotional processes. For instance, Wiswede et al. (2009) have found that a transient induction of negative affect enhanced the error-related negativity measured by EEG and Van der Borght et al. (2016) found that PES is adaptive for individuals low in anxiety, but relates to a non-adaptive process in individuals high in trait anxiety. Furthermore, a recent study showed that post-error changes in RT were influenced by emotional properties of the error stimulus (Caudek et al., 2015), and Verbruggen et al. (2016) showed that PES can depend on the context in which the error occurs, where response times after gambling losses were shorter than after winning (unlike PES). Follow-up experiments pointed to an impulsive and non-adaptive underlying cause, rather than a cognitive one.

That the study of post-error processing can reveal the specific influence of emotion on adaptive processes presents an interesting alternative approach when exploring the influence of emotion on visual attention in general. Indeed, despite the fact that there is now a large literature on using facial emotional stimuli in visual search tasks going back almost three decades, the field has been troubled by difficulties in separating the influence of emotional factors on search behavior from the influence of perceptual factors (for an overview, see e.g., Lundqvist et al., 2015). Hence, an approach that moves away from analyzing RTs and accuracy on successful responses and focuses on post-error effects may offer new insights into understanding how emotional stimuli influence our behavior.

Adaptive post-error adaptations have recently been identified in a visual search experiment (Steinhauser et al., 2017). In the current study, we use the proposed model by Ullsperger and Danielmeier (2016) to explore whether this framework of adaptive and non-adaptive PES components can be applied to results from a visual search paradigm consisting of neutral, angry, and happy faces with three levels of difficulty. We then extend the study of drift diffusion parameters to include five consecutive responses after the error to explore the impact of an error on forthcoming decisions. Furthermore, we examine the influence from the emotional stimulus on the error-monitoring process to study how the emotional valence of an error trial influences latent decision processes on the first post-error trial. Finally, we investigate how different decision components on the first post-error trial contribute to accuracy improvements on the following trials.

## Materials and Methods

### Participants

In total, 6,047 participants (2,935 women) took part in the experiment. The experimental setup was at display at the art exhibition “Passions” at Nationalmuseum, Stockholm, Sweden, March 8th to August 12th, 2012, and the data analyzed have been collected from visitors who volunteered to do the task. Apart from gender, no other information about participants was collected. Institutional review board approval was not required for this study as per our institution’s guidelines and national regulations. Consent was implied by participants voluntarily initiating the task after detailed information about the aims of the research was provided to participants, both on the text panels accompanying the installation, and on the TV screen before the experiment began.

### Apparatus

The experiment was programmed using Adobe Director 11 software (Adobe Inc.), run on a Pentium IV computer, with a 60 inch LED TV at a 1024 by 768 pixels resolution and viewed from approximately three meters distance.

### Stimuli

The emotional facial stimuli were selected from the AKDEF dataset (Lundqvist and Litton, 1998). These stimuli consist of images of an averaged male and an averaged female face, each displaying three different expressions (neutral, happy, and angry). The original angry and happy faces were modified to produce emotional expressions at intensities varying between 80 and 100%. The modification of the faces was made with Sqirlz Morph 2.1^1^, and key points were used to guide the morph between facial features (e.g., lips, mouth shape, eyes, nose wrinkles, facial outline). Faces with 80, 90, and 100% emotional intensity were created in the neutral-to-angry and neutral-to-happy continua for the female and male averaged faces, respectively. The morphed faces reflect varying difficulty levels and we refer to the levels of difficulty as I_80_, I_90_, and I_100_, respectively, with I_80_ being the most difficult one to distinguish from neutral. The size of each face was 150 × 200 pixels.

### Visual Search Task

**FIGURE 1:**
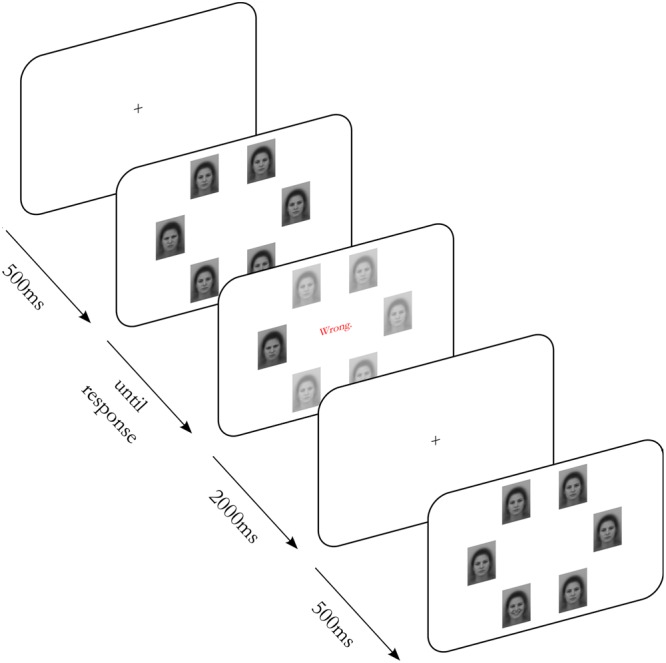
Visual search paradigm. On every trial, six morphed faces from the Averaged Karolinska Directed Emotional Faces dataset (Lundqvist and Litton, 1998), used with consent from the copyright holder, were presented in a circular display. Participants had to decide whether all faces displayed the same emotion (“all neutral”) or one face showed a different emotion (“one deviant”). Angry and happy faces were used as deviant emotions. Feedback (“Correct!” or “Wrong.”) was displayed after a choice and in the case of a missed deviant emotion, that particular face was highlighted.

During the visual search task (**Figure 1**), each stimulus display consisted of six faces, presented in a circular display. In 40% of the displays, the so-called “no target” conditions, all faces were of the same emotional expression (neutral). In 60% of the displays, the so-called “target present” conditions, one of the six faces was of a different emotional expression from that of the background distractors (angry or happy). A target face could occur at any of the six positions, resulting in a total of 12 (2 distractor emotions ^∗^ 6 positions) different displays containing a target, and one display type without target. The gender of presented faces was randomized between participants.

Initial self-paced instructions explained that the task was to decide whether all faces in a display were similar (and then the left key should be pressed with the left index finger), or if one face was different from the others (the right key should be pressed with the right index finger). A trial was initiated by a fixation point presented for 500 ms at the center of the screen. The stimulus display was then exposed until the participant responded, after which a two second feedback message was presented (“Correct!” or “Wrong.”) during which the target face was highlighted, before the fixation point reappeared on the screen, initiating a new trial (RSI: 2,500 ms). Each participant was exposed to a minimum of 20 randomly ordered trials with the same face intensity (difficulty level) on all trials. The experiment ended when participants reached a criterion of 20 correct trials. Error trials were recycled and presented again at a later randomized point among the remaining trials.

### Data Exclusion

We excluded participants who performed with an accuracy of at least three standard deviations below the mean and participants with any single trial above 10 s since this likely reflects little engagement with the task. After exclusion, 5,814 participants (2,868 women) remained in the analysis. Furthermore, we discarded all trials with a log-transformed RT of two standard deviations above or below mean log-transformed RT to constrain analyses to a reasonable range of RT, which lead to removal of about 5% of all trials (Ratcliff, 1993). Thus in total, we analyzed 121,915 trials (110,601 correct trials, 11,314 errors).

### Statistical Analysis

The data were analyzed within R (R version 3.3.0, R Core Team, 2016). We used (generalized) mixed level model analyses with subjects as random effects using the linear mixed-effects models R package *lme4* version 1.1-12 (Bates et al., 2015) and maximum likelihood estimation. For linear mixed models, we used the R package *lmerTest* version 2.0-30 (Kuznetsova et al., 2015) to conduct *F*-tests and Sattherwaite’s approximations to the degrees of freedom and likelihood ratio tests for generalized mixed models. *Post hoc* pairwise tests (single-step method) were implemented with the R package *multcomp* version 1.4-5 (Hothorn et al., 2015) and we report corrected *p*-values where appropriate.

As we were interested in overall effects across task difficulties, we controlled for the effects of difficulty by including it as an independent variable in the statistical models.

#### Reaction Times

To control for potential effects of RT of different trial types on PES we calculated PES by subtracting trial type specific post-error RTs from associated same-trial pre-error RTs (ΔRT, see Dutilh et al., 2012a) and report these results in addition to the post-error RT.

#### Accuracy

We tested whether difficulty level and trial type (Angry, Happy, Neutral) had an influence on accuracy and also tested if accuracy was related to RTs on a trial-by-trial level. Similarly, we examined if accuracy was systematically related to RTs after errors in particular. Finally, we investigated whether accuracy was influenced by the previous emotion on an error trial (happy faces against angry faces).

### Drift Diffusion Analysis

**FIGURE 2:**
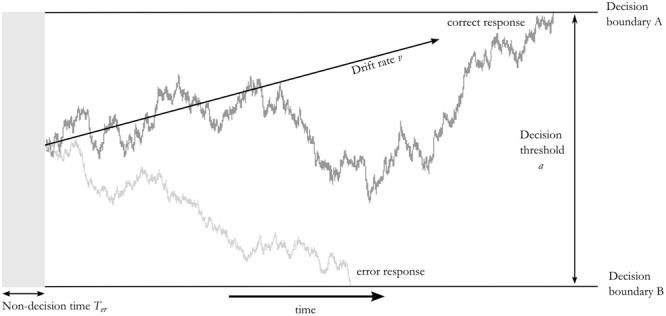
Schematic of the drift-diffusion model. Random walks of two example decisions along the three critical diffusion parameters used in this study are displayed. Evidence is accumulated in a noisy fashion with a drift rate *v* until it reaches one of the two decision boundaries. The distance between the two boundaries is determined by the decision threshold *a*. Non-decision processes before (e.g., early visual processing) and after (e.g., motor execution, not shown) the decision are reflected in the non-decision time *T_er_.*

To investigate decision processes underlying the RTs, we used drift diffusion modeling as implemented in the Hierarchical Drift Diffusion Modeling toolbox (HDDM version 0.6.0, Wiecki et al., 2013) in Python 2.7 (see **Figure 2** for a conceptual overview). In all analyses, we set up seven models related to three central parameters in the drift diffusion model: the decision threshold (*a*), the drift rate (*v*) and the non-decision time (*T_er_*). The seven models were [*a*], [*v*], [*T_er_*], [*a,v*], [*a,T_er_*], [*v,T_er_*], [*a,v,T_er_*]. Among these models, the best-fit model was determined by model comparison using the Deviance Information Criterion (DIC, Spiegelhalter et al., 2002), which takes into account the likelihood of the model and number of parameters modeled, i.e., the complexity of the model, with lower DIC values indicating better fit. It should be noted that DIC values for the second best model fit were in many cases similar to the best model fit. Directions of differences in parameter estimates in these second best models were all concordant with the best fitting model. For the modeled parameters, we used the default priors implemented in HDDM, based on previous studies as collected by Matzke and Wagenmakers (2009).

Convergence of chains was assessed through visual inspection and use of the Gelman Rubin convergence statistic (Gelman and Rubin, 1992). For all diffusion models, we included only participants who committed at least one error.

Drift diffusion model chains did not converge properly when estimating parameters on individual participant level. This was likely due to individual participants having a very low absolute amount of errors (1.72 CCEx error trials per participant on average in the final models). We therefore report all drift diffusion parameters estimated on group level. However, it should be noted that the parameters for single subject models indicated effects into the same direction as the group based models for all parameter estimates besides non-decision time estimates for two models.

For each of the final models, we generated 50,000 samples from the joint posterior distribution of all respective parameters using Markov chain Monte Carlo sampling (Gamerman and Lopes, 2006) and discarded the initial 10,000 samples as burn-in.

#### Model 1: First Trial after an Error

First, we implemented a model that captures the decision process on the trial after an error, by comparing these trials to those that are preceded by at least three correct trials. To avoid processes biased by several consecutive errors we also constrained error trials to be preceded by at least two correct responses (CCEx: *n* = 6,195, CCCx: *n* = 41,407). To investigate whether difficulty of trials had an effect on the decision process we added a model that estimated separate parameters for the three difficulty levels used in the task, separately for post-error and post-correct trials.

#### Model 2: Trials 2–5 after an Error

Next, we ran models for trials with a distance of two to five trials following the error or correct trial to investigate whether an error had a lasting effect on the decision process several trials ahead. For this analysis, all intervening trials were required to be correct (two trials in between: *n* = 34,191; three trials in between: *n* = 24,848; four trials in between: *n* = 18,439; five trials in between: *n* = 13,521).

#### Model 3: First Post-error Trial Predicting Accuracy on Following Trials

Third, to isolate components of the decision process of the first post-error trial that facilitate more accurate responses in the future, we compared post-error trials that were followed by five correct trials (*n* = 2,476) with those that were followed by one or more errors on the next five trials (*n* = 1,679).

#### Model 4: First Post-error Trial, Divided by Error on Angry or Happy Trials

Finally, we specified a model comparing first post-error trials depending on whether the error was committed when an angry (*n* = 3,905) or when a happy (*n* = 1,325) face was shown.

## Results

### Reaction Times

**FIGURE 3:**
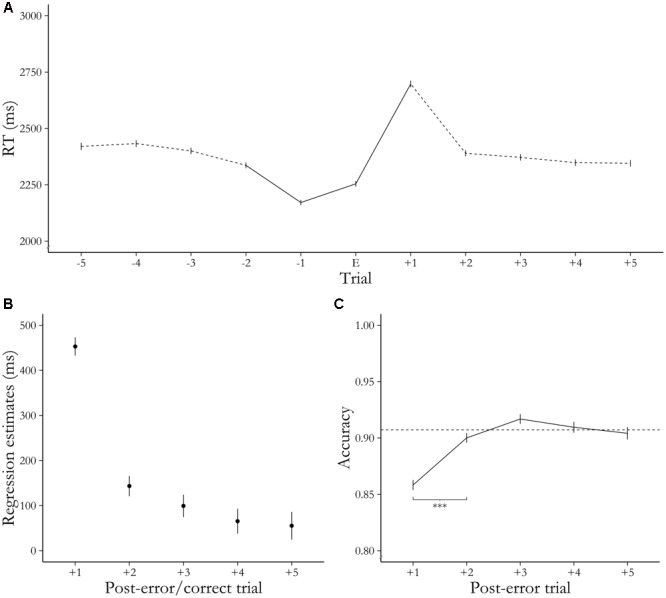
RT and accuracy results. **(A)** Average RTs on trials pre- and post-error. All trials besides the error are required to be correct trials. Bold line indicates minimal sequence of analysis for an error (CCEC). Error bars represent SEM. **(B)** Beta estimates indicating the difference between post-error and post-correct trials in RT, depending on distance to error/correct. Error bars represent 95% CI. **(C)** Average accuracy on post-error trials. Dotted line displays average accuracy over the whole task. Error bars represent SEM. ^∗∗∗^*p* < 0.001.

Participants slowed their response time on the first trial after errors compared to after correct responses, as demonstrated both by RT [*b* = 452.96, *t*(75934) = 43.81, *p* < 0.001, **Figure 3A**] and ΔRT [*b* = 583.41, *t*(21491) = 27.34, *p* < 0.001]. This was also the case for the subsequent trials 2–5 after the error, even though regression coefficients indicated a progressively smaller difference between post-error and post-correct trials over time [**Figure 3B**; Dist2 RT: *b* = 143.42, *t*(58135) = 12.47, *p* < 0.001, Dist2 ΔRT: *b* = 327.12, *t*(20135) = 13.68, *p* < 0.001, Dist3 RT: *b* = 99.36, *t*(55630) = 7.79, *p* < 0.001, Dist3 ΔRT: *b* = 281.07, *t*(15985) = 10.25, *p* < 0.001, Dist4 RT: *b* = 65.41, *t*(42449) = 4.66, *p* < 0.001, Dist4 ΔRT: *b* = 245.79, *t*(13389) = 8.36, *p* < 0.001, Dist5 RT: *b* = 55.34, *t*(37599) = 3.54, *p* < 0.001, Dist5 ΔRT: *b* = 247.60, *t*(11809) = 7.20, *p* < 0.001].

Errors on trials showing an angry face among the neutral faces lead to PES on first trial after error of larger magnitude than happy faces [RT, *b* = 69.63, *t*(4567) = 2.01, *p* = 0.044, ΔRT, *b* = 132.54, *t*(1414) = 2.32, *p* = 0.020]. This effect persisted when we constrained the analysis of the post-error trial to only neutral trials [RT, *b* = 151.95, *t*(1557) = 2.66, *p* = 0.008, ΔRT, *b* = 319.53, *t*(488) = 3.56, *p* < 0.001].

### Accuracy

Accuracy was lower on the first trial after an error than after correct trials [χ^2^(1) = 77.42, *p* < 0.001; *b* = -0.395, *z* = -9.02, *p* < 0.001; average accuracy post-correct: 92.6%, average accuracy post-error: 85.8%]. After Bonferroni correction for multiple comparisons, there was no significant difference in individual comparisons for post-error against post-correct trials at any of the four subsequent distances investigated [Dist2 Acc: χ^2^(1) = 0.299, *p* = 0.585, Dist3 Acc: χ^2^(1) = 4.596, *p* = 0.032, Dist4 Acc: χ^2^(1) = 0.26, *p* = 0.608, Dist5 Acc: χ^2^(1) = 4.49, *p* = 0.034]. We found that accuracy increased over time after the error [**Figure 3C**, χ^2^(4) = 86.58, *p* < 0.001] and this increase was driven mainly by trials after the initial post-error dip in accuracy (E+2 > E+1: *z* = 6.18, *p* < 0.001; E+3 > E+2: *z* = 2.46, *p* = 0.051; E+4 > E+3: *z* = -1.35, *p* = 0.487; E+5 > E+4: *z* = -0.98, *p* = 0.745).

Further, accuracy differed slightly between the three difficulty levels [χ^2^(2) = 46.86, *p* < 0.001; average accuracy I_100_: 91.6%, average accuracy I_90_: 90.8%, average accuracy I_80_: 89.7%; I_100_ > I_90_: *z* = 2.82, *p* = 0.013, I_100_ > I_80_: *z* = 6.81, *p* < 0.001, I_90_ > I_80_: *z* = 3.97, *p* < 0.001] and between the three different trial types [χ^2^(2) = 4127.8, *p* < 0.001; average accuracy all neutral face trials: 95.5%, average accuracy happy face trials: 93.3%, average accuracy angry face trials: 82.9%; neutral > happy: *z* = 13.10, *p* < 0.001, neutral > angry: *z* = 56.28, *p* < 0.001, happy > angry: *z* = 41.98, *p* < 0.001]. Accuracy on the post-error trial did not significantly differ between previous errors on angry trials and happy trials [angry: 85.7%, happy: 87.1%, χ^2^(1) = 1.48, *p* = 0.224].

### Relationship between Accuracy and Reaction Times

We found a general relation between z-scored RT and accuracy on the first post-correct trial [*b* = 0.1499, χ^2^(1) = 85.19, *p* < 0.001] but there was no significant relationship between *z*-scored RT on the first post-error trial and accuracy [*b* = -0.03, χ^2^(1) = 0.6893, *p* = 0.406]. RTs on the first post-error trial were, however, associated with average accuracy on the five subsequent trials [*b* = 753.08, *t*(3544) = 7.495, *p* < 0.001].

### Drift Diffusion Analysis

#### Model 1: First Trial after an Error

**FIGURE 4:**
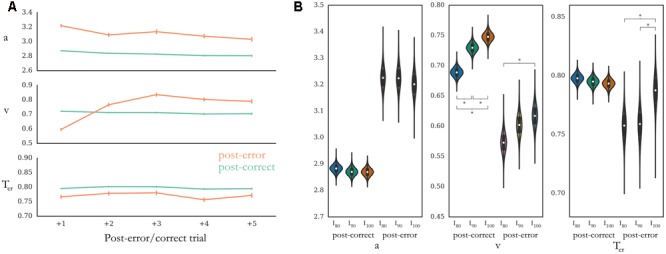
HDDM parameter estimates for post-error and post-correct trials. *a*, decision threshold; *v*, drift rate; *T_er_*, non-decision time. **(A)** Posterior estimates of HDDM parameters by distance from error and correct trial, respectively. Error bars represent standard deviation of posterior estimate. Bayesian probability for posterior differences of all comparisons between post-error and post-correct trials was larger than 95%. **(B)** Violin plots for posterior estimates of HDDM parameters for the decision process on post-error against post-correct trials at distance 1, separately for the three difficulties (80 denotes the hardest, 100 the easiest difficulty) and diffusion parameters. Bayesian probabilities for posterior differences greater than 95% are marked with a “^∗^”. Note that this denotation is different to the Frequentist probability of a *p*-value.

**FIGURE 5:**
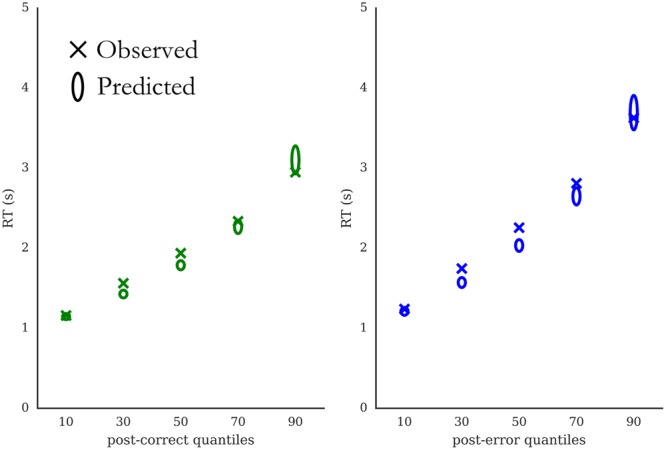
Posterior predictive checks. Quantile probability plots for post-correct trials **(Left)** and post-error trials **(Right)**, respectively. Crosses mark reaction time means of five quantiles (10, 30, 50, 70, and 90%). Ellipses indicate quantile mean RTs predicted by the model. Height of ellipses corresponds to standard deviation of posterior predictive distribution for respective quantile.

Among the diffusion models tested, a model with decision threshold, drift rate and non-decision time as freely varying parameters provided the best fit when comparing the first trial after an error and the first trial after a correct response (see **Table 1** for DIC values). The first trial after an error was characterized by an increase in decision threshold and a simultaneous decrease both in drift rate and non-decision time (**Figure 4A** and **Table 2**). Posterior predictive checks indicated that the modeling could account for RT distributions observed in the task in relation to the two conditions (**Figure 5**). Manipulations of task difficulty had the strongest effect on the drift-rate *v*, with easier difficulties leading to a higher drift rate. This effect was most pronounced for post-correct trials [Bayesian posterior probabilities: P*_v_*(PC I_100_ > I_80_) = 100%, P*_v_*(PC I_100_ > I_90_) = 95.14%, P*_v_*(PC I_90_ > I_80_) > 99.99%; P*_v_*(PE I_100_ > I_80_) = 96.74%, P*_v_*(PE I_100_ > I_90_) = 72.31%, P*_v_*(PE I_90_ > I_80_) = 89.09%, **Figure 4B** and **Table 3**]. Furthermore, we found a higher non-decision time for post-error trials between highest difficulty and both medium [P*_t_*(PE I_100_ > I_90_) = 95.03%] and lowest difficulty [P*_t_*(PE I_100_ > I_80_) = 96.08%]. None of the other parameter differences surpassed a threshold of 95%.

**Table 1:** Relative DIC to best fitting model.

Model	*a*	*v*	*T_er_*	*a, v*	*a, T_er_*	*v, T_er_*	*a, v, T_er_*
Distance 1	152.12	237.18	507.18	14.41	133.68	214.63	w
Distance 2	19.91	83.71	76.82	8.16	13.51	78.45	w
Distance 3	62.15	99.43	104.45	4.61	60.95	86.2	w
Distance 4	52.31	56.8	68.88	20.07	37.17	58.75	w
Distance 5	17.88	36.19	37.32	2.22	16.88	34.64	w
Accuracy	19.47	65.78	63.89	4.36	15.65	62.29	w
Emotion	4.38	w	6.91	1.5	4.02	1.28	1.36

*Smaller DIC values indicate better fit. Best fitting models are denominated with a “w”.*

**Table 2:** Drift diffusion model parameter estimates for distances 1–5 to an error/correct trial.

Measure	Distance 1	Distance 2	Distance 3	Distance 4	Distance 5
*a*(PC)	2.874	2.848	2.828	2.811	2.82
	*P* = 0	*P* = 0	*P* = 0	*P* = 0	*P* = 0
*a*(PE)	3.219	3.09	3.136	3.087	3.055
*v*(PC)	0.721	0.713	0.712	0.708	0.72
	*P* = 1	*P* < 0.01	P = 0	*P* = 0	*P* = 0
*v*(PE)	0.596	0.766	0.836	0.815	0.802
*T_er_*(PC)	0.795	0.802	0.801	0.803	0.804
	*P* > 0.99	*P* > 0.99	P > 0.99	*P* = 1	*P* = 0.984
*T_er_*(PE)	0.767	0.778	0.780	0.763	0.784

*Parameter values correspond to **Figure 4A**. *P*-values below post-correct parameters indicate Bayesian probability of a larger posterior estimate for post-correct trials compared to post-error trials. PC = post-correct. PE = post-error.*

**Table 3:** HDDM parameter estimates for distance 1 by difficulty.

Measure	Post-correct			Post-error		
	I_80_	I_90_	I_100_	I_80_	I_90_	I_100_
*a*	2.883	2.87	2.869	3.226	3.224	3.202
*v*	0.689	0.73	0.748	0.573	0.602	0.617
*T_er_*	0.798	0.795	0.793	0.757	0.759	0.787

#### Model 2: Trials 2–5 after an Error

Diffusion analyses for trials subsequent to the first trial after errors (trials 2–5) showed a sustained increase in decision threshold and decrease in non-decision time, similar to the first trial after the error. However, the initially lowered drift rate increased after the first post-error trial and reached a higher level than after post-correct trials (**Figure 4A** and **Table 2**).

#### Model 3: First Post-error Trial Predicting Accuracy on Following Trials

**FIGURE 6:**
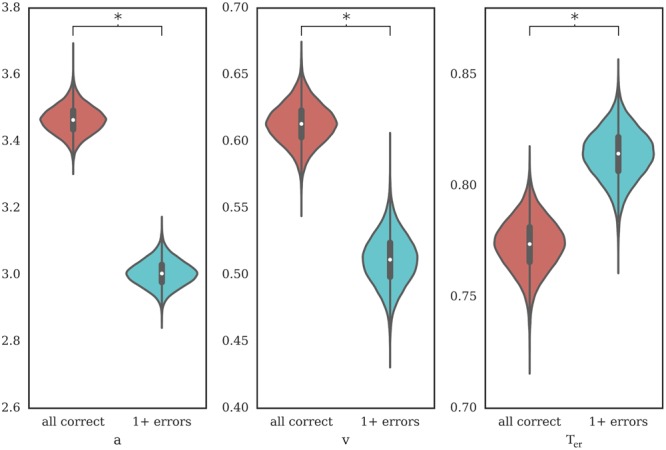
Posterior estimates of HDDM parameters on first trial after an error, comparing trials with five subsequent correct trials against those with at least one error. Bayesian probabilities for posterior differences greater than 95% are marked with a “^∗^”. *a*, decision threshold; *v*, drift rate; *T*_er_, non-decision time.

A drift diffusion model letting all three parameters vary provided the best fit to the data as measured by DIC (**Table 1**). We found a larger decision threshold (Bayesian posterior probability: 100%) and higher drift rate (Bayesian posterior probability: 100%) as well as shorter non-decision time (Bayesian posterior probability: 99.4%) on the first post-error trials which were followed by only correct trials compared to the ones followed by one or more errors (**Figure 6**).

#### Model 4: First Post-error Trial, Divided by Error on Angry or Happy Trials

**FIGURE 7:**
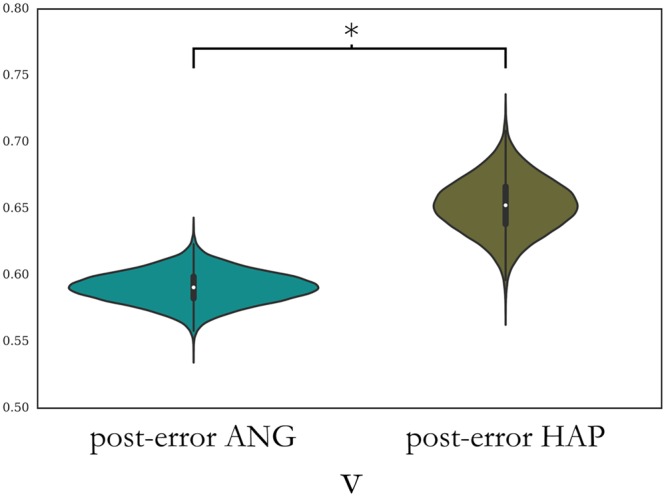
Posterior estimates of HDDM parameters on first trial after an error, comparing previous errors on angry faces against happy faces. Bayesian probability for posterior difference greater than 95% is marked with a “^∗^”. *v*, drift rate.

Model comparisons (**Table 1**) suggested that the best fit was attained for the model which included only the parameter for the drift rate (*v*) when comparing first post-error trials by the error trial type. Errors on angry face trials resulted in a lower drift rate (Bayesian posterior probability of the drift rate being lower after errors on angry faces: 99.56%, **Figure 7**).

## Discussion

Our results (see **Table 4** for an overview) show that after errors are made on a visual search task, response time slows down and response accuracy deteriorates on the first trial post-error. Errors hence entail a cost. The results also show that this cost is influenced by the error context, where errors on angry face trials lead to PES of larger magnitude compared to errors on happy face trials. We further demonstrate that the PES magnitude on the first trial after an error influences future (five subsequent trials), but not immediate (first trial after the error), response accuracy. PES on the first trial after an error therefore appears to reflect both the impact of the past error, and an adaptation toward future trials.

**Table 4:** Behavioral and modeling results.

Measure	First post-error trial	Post-error trials 2–5
Reaction time	Slowing (enhanced slowing for angry compared to happy errors)	Progressive decrease in slowing
Accuracy	Decrease	No significant difference
Reaction time and accuracy	No significant relation	Long RTs on first post-error trial associated with higher accuracy on the next five trials
**Decision process**		
*Decision threshold*	Increase (higher decision threshold related to higher accuracy on trials 2–5)	Increase
*Drift rate*	Decrease (stronger decrease for angry than happy errors; higher drift rate related to higher accuracy on trials 2–5)	Increase
*Non-decision time*	Decrease	Decrease

*All reported measures refer to comparisons of post-error versus post-correct trials.*

### Post-error Decision Making Is Marked by a Sustained Increase in Decision Threshold and a Transient Decrease in Evidence Accumulation

From drift diffusion modeling of our data we provide further support for the notion that response time adaptation after errors is a multi-component phenomenon, synthesizing both functional and non-functional accounts of PES (Purcell and Kiani, 2016; Steinhauser et al., 2016; Van der Borght et al., 2016). Our models show that errors during the visual search task were followed by an increase in decision threshold that remained above post-correct decision threshold levels over the course of the following five trials. This result indicates that the error prompts not only an immediate adjustment in response caution, but also leads to subsequently more cautious decisions. Drift rate initially declined after an error but increased over the following trials, reaching even higher levels compared to post-correct trials. The decline most likely reflects the initial dip in accuracy during the first post-error trial and the subsequent increase reflects the regained accuracy across trials 2–5, a mechanism which could be taken to correspond to task-related decreases and increases in attention as proposed by a recent theoretical account of post-error adjustments (Ullsperger and Danielmeier, 2016).

### Higher Decision Threshold and Drift Rate Immediately Post-error Benefit Subsequent Performance

Both higher decision threshold and evidence accumulation rate on the first trial after an error were associated with better performance on subsequent trials in this task. In drift diffusion models, positive adjustments of these two parameters usually reflect higher response accuracy (Cavanagh et al., 2014; Ratcliff et al., 2016). We find that on the first trial after an error, this relationship also holds for accuracy on future trials (the next five trials) after the error.

This result illustrates how the post-error adaptation process may influence accuracy of future responses (Hester et al., 2007; Schiffler et al., 2016). Given that task-relevant neural activation increases and decreases have previously been found in relation to post-error adaptations (King et al., 2010; Danielmeier et al., 2011; Steinhauser et al., 2017), this finding is not surprising.

### Non-decision Time and Errors

The modeling also shows that errors were associated with small, but sustained decreases in non-decision time on trials after the error. Conceivably, the lower non-decision time found after errors in this task reflects a more vigorous and therefore quicker motor execution. However, whether that is the case or whether it corresponds to quicker encoding of relevant stimuli as shown previously (Rinkenauer et al., 2004) is a question to be explored in future studies, potentially employing neural measurements to corroborate the evidence (Roitman and Shadlen, 2002; Kiani et al., 2008).

### Diffusion Parameters Are Influenced by Task Difficulty and Stimulus-Dependent Error Properties

Model parameters proved to vary consistently with how external factors had been manipulated. The rate of evidence accumulation was faster for easier difficulty levels on both post-error and post-correct trials. Yet, both drift rate and decision threshold differences were larger when comparing post-error to post-correct trials than across difficulties, which shows that endogenous error monitoring had a larger effect on the decision process parameters than external manipulation of difficulty.

The results also demonstrated that the emotional expression of the stimuli on the trial during which the error was made had an impact on the decision process after the error. Errors on angry face trials led to a decrease in evidence accumulation following the error, compared to errors committed on happy face trials. As no increase in accuracy concurred with PES on the first trial after the error, this suggests a distraction from the task induced by the emotional processing of the different facial stimuli. These differences may stem from an attentional re-orienting response when evaluating a potentially threatening situation (as suggested by Ullsperger and Danielmeier, 2016).

In support of such a notion, it has been demonstrated that the emotional valence of facial stimuli directly influences how attention is engaged during visual search tasks such as the one used in this study (Lundqvist et al., 2015). However, the literature also shows that angry faces on average require more time for emotion recognition as compared to happy faces (Nummenmaa and Calvo, 2015). Hence, the question of whether the increased PES caused by errors on angry faces stems from effects on how attention is engaged by emotional valence, or from differences in the processing speed during emotion recognition which affect the drift rate on next trial after the error needs to be explored in future studies.

In a similar visual search paradigm to ours, Caudek et al. (2015) found (unlike us) that errors on angry face trials led to response time speeding on the next trial. The discrepancies in results could possibly reflect differences in how aware participants were of their mistake, since in the study of Caudek et al. (2015), no explicit feedback was provided to participants after they made a decision. Although error detection exists in the absence of explicit feedback (Hester et al., 2005; Bengtsson et al., 2009), PES has been more consistently observed after perceived compared to unperceived errors (Nieuwenhuis et al., 2001; Wessel et al., 2011). It could also be that the discrepancies in results depend on differences between the particular emotional stimuli used since stimulus selection and between-expression intensity differences have been demonstrated to strongly influence the direction of results when comparing angry and happy faces in visual search tasks (Lundqvist et al., 2014).

### Limitations

While this study investigated adaptive and non-adaptive reactions to an error in a large pool of participants, the low amount of trials per participant implies that the distribution of errors is skewed toward initial encounters of errors rather than a repeated error reaction. Future studies should investigate whether the results obtained here generalize to errors made in later phases of a task after the initial adaptations to an error. Furthermore, future experiments could also address whether it is possible to influence the different post-error decision components, e.g., by providing varying error feedback that selectively impacts on decision threshold adaptations or evidence accumulation changes. Another interesting question that could be investigated in the future is whether individual differences exert a specific influence on time courses of post-error adaptations as for instance suggested by a recent study relating trait anxiety to post-error accuracy (Van der Borght et al., 2016).

## Conclusion

Taken together, our findings in a large scale visual search experiment elucidate the time courses of both adaptive and non-adaptive decision components of PES. That PES consists of non-adaptive as well as adaptive components is in line with recent findings (Steinhauser et al., 2016; Van der Borght et al., 2016).

We demonstrated that errors evoked a sustained increase in response threshold that lasted for several trials, suggesting a stable rise in response caution. In parallel, errors also entailed a transient decrease in evidence accumulation with a gradual increase over the following trials. This indicates a disadvantageous immediate impact of the error and a potential subsequent increase in attention, corresponding to accuracy returning to average levels.

These findings resonate with recent empirical findings in primates (Purcell and Kiani, 2016) and theoretical outlines on post-error adaptations (Ullsperger and Danielmeier, 2016). Further, our results suggest that emotional stimulus-specific information from the error trial affects post-error adaptation, and primarily seems to influence the attentional component, which is in line with the findings of Van der Borght et al. (2016). Finally, more accurate future trials could be differentiated from less accurate trials through latent process components of the decision during the first post-error trial. This shows that even in the absence of a direct relation of RT and accuracy on the post-error trial, post-error adaptations could still be beneficial with regard to future trials, as has been previously observed (Schiffler et al., 2016).

Our results show the importance of not only focusing on the immediate post-error trial but also investigating the medium- to long-term effects of an error on latent decision processes using model-based approaches in order to improve the understanding of how learning takes place.

## Author Contributions

BS conceived the idea for the study. DL contributed to the experimental design and data acquisition. BS analyzed the data. BS, SB, and DL contributed to the interpretation of the data. BS, SB, and DL drafted the manuscript and provided critical revisions. BS, SB, and DL approved the manuscript version to be published, and agreed to accountability for all aspects of the work.

## Conflict of Interest Statement

The authors declare that the research was conducted in the absence of any commercial or financial relationships that could be construed as a potential conflict of interest.

## Footnotes

1 http://www.xiberpix.net
